# Evaluating buffering effects for social determinants of health and type 2 diabetes in the All of Us Research Program

**DOI:** 10.21203/rs.3.rs-6315181/v1

**Published:** 2025-05-07

**Authors:** Vincent Lam, Shivam Sharma, Christian S. Alvarez, I. King Jordan, Leonardo Mariño-Ramírez

**Affiliations:** National Institutes of Health; Georgia Institute of Technology; National Institutes of Health; Georgia Institute of Technology; National Institutes of Health

**Keywords:** Health disparities, diabetes, interactions, All of Us, social determinants of health

## Abstract

**Introduction:**

Social determinants of health (SDOH) play a strong role in influencing type 2 diabetes (T2D) risk. Certain protective SDOH have been demonstrated to mitigate or buffer against the positive associations between risk-associated SDOH and poor health outcomes, though such buffering effects have only been evaluated among a limited range of SDOH and outside the context of T2D. This study aims to assess potential buffering effects among SDOH associated with T2D case status.

**Methods:**

The study population was constructed from *All of Us* Research Program data. Survey data were used to derive composite metrics measuring 10 different SDOH. Logistic regression models modeling T2D case status as a function of each SDOH were used to designate each as either a T2D risk-associated or protective factor. Interaction models were used to measure potential buffering effects between T2D risk-associated and protective factors.

**Results:**

The main study population consists of 215,270 participants. All ten SDOH were significantly associated with T2D. Social support and neighborhood cohesion emerged as T2D protective factors, with the rest being classified as risk-associated factors for T2D. A single buffering effect was observed between social support and stress (β = 0.43, p <0.05) (95% CI, −0.84 to −0.01). Reverse buffering effects were observed between neighborhood cohesion and discrimination, food insecurity, and individual-level socioeconomic deprivation (SED). Reverse buffering effects were also observed between social support and food insecurity, individual-level SED, and spirituality.

**Conclusions:**

The buffering effect observed between social support and stress on T2D is consistent with previous findings. The reverse buffering effects observed in interactions between certain SDOH factors suggest that some risk-associated SDOH may weaken the potential benefits of certain protective SDOH. Interventions aimed at alleviating adverse socioeconomic conditions may have a compounding effect on reducing T2D risk by enabling communities to benefit from T2D protective factors.

## BACKGROUND

In public health research, social determinants of health (SDOH) have been recognized as key factors in shaping health outcomes [1, 2]. SDOH are defined by the World Health Organization as “the non-medical factors that influence health outcomes”. SDOH encompass factors related to the conditions and environments in which people live and work. They can include but are not limited to one’s employment status, education, housing, healthcare access, and social inclusion [3]. SDOH strongly influence health, with such factors being estimated to contribute to 30–60% of all health outcomes [3–5]. For instance, individuals who are more highly educated and who have higher incomes are at reduced risk for conditions such as diabetes and generally have better health and longer lifespans [6–9]. On the other hand, individuals with adverse SDOH exposures such as discrimination and stress often demonstrate worse health outcomes [10–13].

Different SDOH often act in concert to influence health outcomes. Individuals exposed to harmful working conditions are more likely to live in poorer environments due to the typically low wages associated with such jobs [14]. Furthermore, synergistic effects on poor health outcomes have been observed between SDOH such as economic hardship and social capital [15]. In other instances, certain SDOH have been shown to mitigate or buffer the effects of adverse SDOH on health. One of the most notable examples can be found in the stress-buffering hypothesis, whereby social support weakens the positive association between stress and negative health outcomes [16, 17]. Similar effects have been observed in other SDOH, with resilience having previously been shown to buffer the positive association between discrimination and poor mental health outcomes [18, 19].

Type 2 diabetes (T2D) is a potentially compelling subject disease for the study of how different SDOH buffer the detrimental effects of other SDOH on health outcomes. T2D is a complex common disease that comprises roughly 90% of the 38.4 million cases of diabetes in the US [20, 21]. Its etiology is associated with a combination of genetic and environmental factors, the latter including various SDOH [22–24]. Stress is among the SDOH shown to increase risk of T2D, potentially through the activation of biological responses commonly implicated in the disease [25] [26]. As social support has been shown to buffer the effects of stress on health outcomes, there is a possibility that social support and other similar SDOH protective factors may reduce T2D risk through the buffering of stress and other T2D risk-associated factors [16, 17]. Buffering effects on risk-associated factors for T2D-related symptoms have been previously explored in literature, though such explorations have mainly been in the context of T2D distress (i.e. the emotional burden of living with T2D) [27–29]. There have been few studies on how SDOH specifically buffer the effects of adverse SDOH on T2D risk. As such, the goal of this study is to explore the potential buffering role of protective SDOH by examining their modifying effect on the associations between risk-related SDOH and T2D.

## METHODS

### Study Population

The study population was constructed using participant data available through the *All of Us* Researcher Workbench—a cloud computing platform through which registered users can access and interact with data from the *All of Us* Research Program. The *All of Us* participant body consists of adults aged 18 and older residing in the US or in a US territory. Volunteers can enroll in the program online via JoinAllofUs.org or in person through a participating healthcare provider. Individuals who are incarcerated or unable to provide consent are not eligible to enroll in the program.

Participant demographic, electronic health record (EHR), and survey data were drawn from the *All of Us* Registered Tier Dataset v7 (curated version R2022Q4R9). Enrollment began on May 31st, 2017, with data collected up to July 1st, 2022. Extracted demographic data consisted of participant date of birth and assigned sex at birth. International Classification of Diseases codes (ICD-9-CM and ICD-10-CM) were extracted from participant EHR data and mapped to phecode 250.2 to classify participants as T2D cases and non-cases [30]. Patients with phenotypes corresponding to phecodes 249–250.99 were excluded from analyses following phecode exclusion criteria. The study sample was thus restricted to individuals who have EHR data available and who were classified as either T2D cases or non-cases. Furthermore, the study sample was restricted to individuals who were assigned either male or female at birth in order to facilitate the use of sex as a covariate in study models.

### Measuring Social Determinants of Health

Social determinants of health were measured using data collected from the *All of Us* “Social Determinants of Health” survey, which includes questions from previously validated surveys and scales. Composite metrics for eight SDOH were constructed using responses to these surveys, with each metric representing a distinct social determinant, e.g.: (1) everyday discrimination, (2) discrimination in healthcare settings, (3) food insecurity, (4) neighborhood cohesion, (5) perceived neighborhood disorder, (6) social support, (7) spirituality, and (8) stress, with this terminology derived from the source material of each question set. Participants can provide responses ranging from “strongly disagree” to “strongly agree” or from “never” to “all the time” based on the question asked. These responses were coded as ordinal values starting from 0 and incrementing by 1, with greater values representing greater degrees of the SDOH.

Matrices consisting of participant ordinal responses to different questions were generated for each of the eight question sets. As they were comprised of ordinal values, these matrices were converted to eight polychoric correlation matrices using the polychoric function from version 2.4.1 of the psych package in R version 4.3.1. The *eigen* function was used to perform principal component analysis on each of the polychoric correlation matrices. This function generated eight new matrices composed of eigenvectors (principal components) arranged in decreasing order of their corresponding eigenvalues. The first principal component of each matrix was min-max normalized and used as a metric for each of the eight social determinants of health. These composite metrics had values ranging from 0 to 1, with greater values representing greater degrees of the social determinant of health they measure.

Socioeconomic deprivation (SED) was measured at both the individuals and zip code levels to capture the multifaceted nature of socioeconomic influences on health. An individual-level metric for SED was derived from participant responses to questions from the *All of Us* “The Basics” survey through methods described by Gupta et al [31]. Variables incorporated into this metric include education, employment, insurance status, home ownership status, and household income. Zip code-level SED was measured using a composite metric created by Brokamp et al [32]. Variables incorporated into this metric include the fraction of households below the poverty level, median household income, the fraction of adults older than 25 with at least a high school education, the fraction of the population without health insurance, the fraction of the population receiving public assistance, and the fraction of homes in the area that are vacant. This metric is available on the Researcher Workbench for most *All of Us* participants. Both measures of SED have values ranging from 0 to 1, with greater values representing more severe levels of SED.

### Statistical Analyses

All statistical analyses were performed using R version 4.3.1. As participants varied in the types of SDOH information they provided, participant datasets used in statistical analyses represent subsets of the main study sample. To classify the ten SDOH as either factor associated with increased or decreased T2D, logistic regression models were used for each social determinant using the *glm* function from the *stats* package. In each model, T2D status (case = 1, non-case = 0) was modeled as a function of an individual SDOH. Participant age and sex were included as covariates to account for potential confounders due to the overrepresentation of older and female participants in the All of Us sample. SDOH with positive and significant association with T2D were classified as risk-associated SDOH, while those with negative and significant associations were classified as potential protective factors.

To examine potential buffering effects, interaction terms between protective and risk-associated SDOH factors for T2D were added to the logistic regression models for all possible combinations of SDOH. Buffering effects were defined as negative and significant interactions, indicating that a protective factor mitigates the effect of a risk-associated factor on T2D. In contrast, positive interactions between SDOH that are protective and SDOH that increase risk indicate that the effects of risk-associated factors mitigate the effects of protective factors on T2D; positive interactions of this kind are referred to as reverse buffering [33]. These tests were supplemented with sensitivity analyses, in which the same interaction analyses were run with composite metric values converted to z-scores. Variance inflation factor analysis was used to assess collinearity between each risk-associated and protective factor pair. A p-value threshold of less than 0.05 was used to determine statistical significance in all analyses.

## RESULTS

### Participant Characteristics

The sample for this study consisted of 215,278 individuals classified as T2D cases or non-cases based on EHR data and assigned sex at birth (Table 1 and Figure S1). Participants were predominantly female and middle-aged or older. The overall prevalence of T2D in the sample was 24.26%.

### SDOH Analyses

Ten SDOH as measured by their respective composite metrics, were tested for the extent to which they were associated with T2D (Table 2). All ten SDOH were found to be significantly associated with T2D (p < 0.05). Of these SDOH, neighborhood cohesion (β = −1.21, p < 0.05) and social support (β = −0.80, p < 0.05) were associated with decreased odds of T2D and were therefore classified as T2D “protective factors”. The remaining eight SDOH were associated with increased odds of T2D and were therefore classified as “risk-associated factor”. These include area and individual-level SED, food insecurity, perceived neighborhood disorder, discrimination, stress, healthcare discrimination, and spirituality. The most potent risk-associated factor for T2D by effect size was area-level SED (β = 2.46, p < 0.05) whereas the least potent factor was spirituality (β = 0.85, p = 1.05e-107). No significant collinearity was observed between any pair of risk-associated and protective factor (Table S1). When not controlling for age or sex, these classifications remain consistent except for stress, which is not significantly (Table S2).

**Figure 1:**
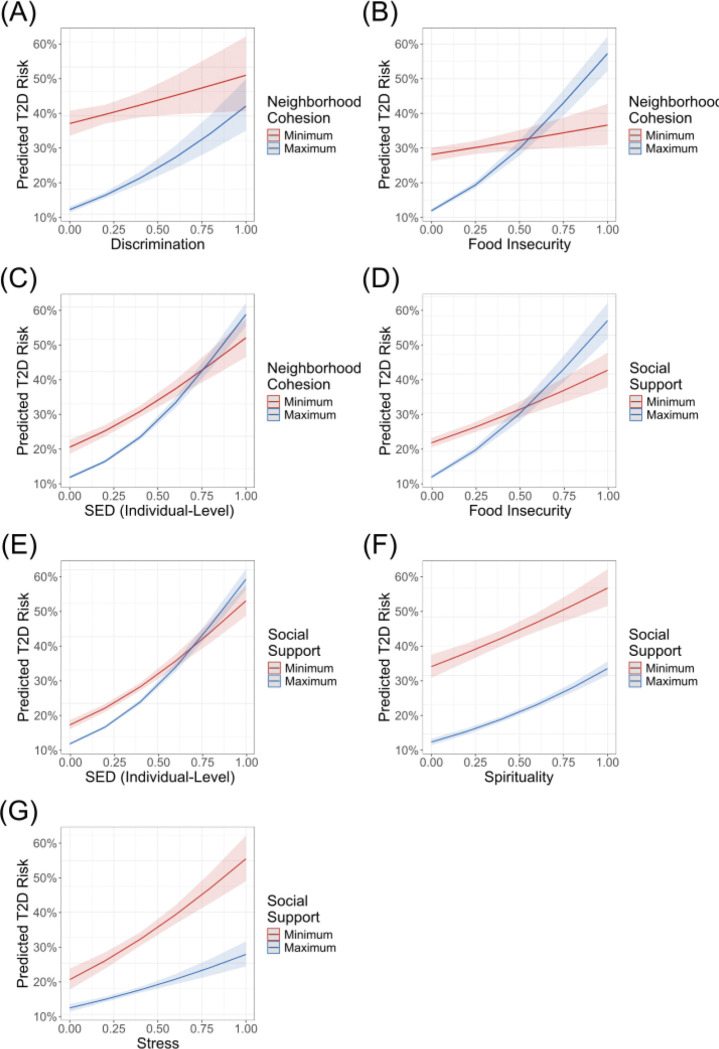
Interactions between T2D risk-associated and protective factors on T2D risk, adjusted for age and sex. Interaction effects between a selected T2D risk-associated factor (x-axis) and protective factor on predicted T2D risk (y-axis) based on the model T2D ~ RF*PF + age + sex, where RF=risk-associated factor and PF=protective factor.

Interaction effects between all combinations of T2D, including protective and risk-associated factors were assessed to identify potential buffering effects (Table 2 and Fig. 1). The first protective factor, neighborhood cohesion, demonstrated positive interactions with food insecurity (β = 1.90, p < 0.05), individual-level SED (β = 1.22, p < 0.05), and everyday discrimination (β = 0.90, p < 0.05). The second protective factor, social support, demonstrated reverse-buffering effects with risk-associated factors such as food insecurity (β = 1.14, p < 0.05), individual-level SED (β = 0.87, p < 4.04e-6), and spirituality (β = 0.37, p < 0.05). Additionally, social support demonstrated a buffering effect with stress (β = −0.43, p < 0.05). When not controlling for age or sex, the reverse buffering effect between neighborhood cohesion and discrimination is no longer observed and a new reverse buffering effect between social support and healthcare discrimination is observed (Table S3 and Figure S2). These significant interactions and their directionality remained when composite metric values were replaced with their corresponding z-scores (Table S4).

## DISCUSSION

In this study, we assessed the potential for various SDOH to exert buffering effects on risk-associated factors for T2D. Of the ten SDOH under investigation, eight emerged as factors associated with T2D and two emerged as T2D protective factors. The classification of each of these SDOH as being either T2D risk-associated or protective factors is largely consistent with their classification in previous literature on T2D epidemiology [25, 26, 34–40]. However, the positive association observed between spirituality and T2D risk appears to be in contrast with previous findings, which suggest that religion and spirituality are generally associated with better health [41]. This association may be driven by individuals using religion or membership to religious communities to cope with the duress brought on by T2D [42, 43]. Furthermore, it may also be driven by unobserved confounding variables that are tightly associated with both spirituality and T2D.

Interaction effects between identified T2D protective and risk-associated factors were assessed to identify potential buffering effects. Several significant interaction effects involving both the neighborhood cohesion and social support protective factors were observed. Of these significant interactions, one buffering effect was observed, which involved social support and stress. This finding is consistent with the stress-buffering hypothesis, suggesting that the buffering effect that social support exerts on positive associations between stress and poor health outcomes may extend to T2D [16, 17]. The remaining six interaction effects observed represent reverse buffering or synergistic effects between the T2D protective factors and corresponding T2D risk-associated factors on T2D. Social support has previously been shown to exert similar reverse buffering effects on positive associations between stress and mental health and on positive associations between workload and job-related stress [44, 45]. Such effects may be explained by the more frequent occurrence of adverse social interactions that may accompany higher degrees of social support. Individuals with higher degrees of social support may also feel a weaker sense of autonomy, which may lead to worse health outcomes [46]. Furthermore, the reverse buffering effects observed may suggest a diminishing return of social support and neighborhood cohesion under adverse conditions. In environments characterized by high SED and food insecurity, social support and neighborhood cohesion may be insufficient to offer substantial protection against the risk of T2D.

These findings are limited as a result of this work being an observational study. The results reported here may have been influenced by a number of unobserved confounding variables. Some such variables may include participant diet and lifestyle, BMI and other comorbidities, which are known to be associated with T2D risk but were not included as covariates in study models [47]. Such variables were not included as covariates due to the lower availability of this data and their potential collinearity with the T2D risk-associated and protective factors presented in the study. Additionally, the *All of Us* participant body is not representative of the US general population. *All of Us* participants are more likely to be female, older, and from underrepresented racial and ethnic groups. The prevalence for many common diseases including T2D is also higher among *All of Us* participants than in the general population [48]. Moreover, the validity of the results presented depend on the composite metrics used and the survey data they are based on, which may not capture all or the most relevant features of the SDOH they were intended to measure. Furthermore, the cross-sectional nature of this study limits the potential to infer causality from its results. Longitudinal studies are needed to validate the results described and to better understand the potential buffering effects of protective SDOH on T2D risk.

## CONCLUSIONS

In conclusion, this study reinforces the known roles of several key SDOH as either T2D protective or risk-associated factors. The buffering effect that social support exhibited on the association between stress and T2D risk supports the stress-buffering hypothesis, suggesting that this hypothesis extends to conditions like T2D. This study also reveals several reverse buffering effects involving the social support and neighborhood cohesion protective factors. These findings may suggest that adverse conditions such as socioeconomic or food deprivation may mitigate the protective effects of social support and neighborhood cohesion on T2D risk. Thus, alleviating such adverse conditions may have a compounding effect on combating T2D risk by further enabling communities to benefit from protective factors.

## Figures and Tables

**Table 1:** Characteristics of *All of Us* participants with EHR data available who were assigned either male or female at birth.

Characteristic	Full Cohort (n = 215,278) (100%)	Female (n = 133,996) (62.24%)	Male (n = 81,282) (37.76%)
**Mean age-years (SD)**	56.44 (16.96)	59.05 (16.73)	54.86 (16.91)
**T2D Cases (Prevalence %)**	52,232 (24.26)	29,992 (22.38)	22,240 (27.36)

**Table 2:** Associations between social determinants of health and T2D risk. T2D ~ SDOH + age + sex

SDOH	Estimate	Std. Error	z-value	p-value
**Risk-associated factors**				
SED (Area-level)	2.46	8.21e-2	29.92	1.10e-196
SED (Individual-level)	2.00	2.22e-2	90.19	0.00e + 00
Food Insecurity	1.65	5.02e-2	32.90	2.15e-237
Perceived Neighborhood Disorder	1.45	6.67e-2	21.76	5.47e-105
Discrimination	1.33	6.98e-2	19.04	8.20e-81
Stress	1.22	6.34e-2	19.23	1.99e-82
Healthcare Discrimination	0.93	6.85e-2	13.64	2.23e-42
Spirituality	0.85	3.85e-2	22.05	1.05e-107
**Protective Factors**				
Neighborhood Cohesion	−1.21	5.92e-2	−20.39	1.86e-92
Social Support	−0.80	4.07e-2	−19.80	3.13e-87

**Table 3:** Interactions between risk-associated factors and protective factors on T2D risk. T2D ~ RF*PF + age + sex, where RF = risk-associated factor and PF = protective factor.

Risk Associated-Factor	Estimate	Std. Error	z-value	p-value
**Protective Factor: Neighborhood Cohesion**				
Food Insecurity	1.90	0.23	8.19	2.53e-16
SED (Area-level)	1.35	0.94	1.43	1.52e-01
SED (Individual-level)	1.22	0.26	4.71	2.52e-06
Discrimination	0.90	0.31	2.92	3.56e-03
Healthcare Discrimination	3.20e-2	0.32	0.10	9.20e-01
Stress	−0.07	0.30	−0.25	8.00e-01
Spirituality	−0.11	0.21	−0.52	6.03e-01
Perceived Neighborhood Disorder	−0.22	0.27	−0.81	4.17e-01
**Protective Factor: Social Support**				
Food Insecurity	1.14	0.17	6.49	8.39e-11
SED (Individual-level)	0.87	0.19	4.61	4.04e-06
SED (Area-level)	0.85	0.67	1.27	2.03e-01
Spirituality	0.37	0.15	2.54	1.12e-02
Healthcare Discrimination	0.25	0.24	1.07	2.84e-01
Discrimination	0.23	0.23	1.02	3.10e-01
Perceived Neighborhood Disorder	0.16	0.23	0.67	5.05e-01
Stress	−0.43	0.21	−2.03	4.28e-02

## Data Availability

All of Us participant data can be accessed and analyzed from the Researcher Workbench by registered users: https://www.researchallofus.org/data-tools/workbench/.

## Abbreviations

BMI: Body mass index
EHR: Electronic health record
SED: Socioeconomic deprivation
SDOH: Social determinants of health
T2D: Type 2 diabetes
US: United States
